# Jacareubin Derivatives Increase Their Anti-Allergic Activity

**DOI:** 10.3390/molecules31101666

**Published:** 2026-05-15

**Authors:** Rosario Tavera-Hernández, Jesabel Pérez-Rodríguez, Antonio Nieto-Camacho, Omar Noel Medina-Campos, José Pedraza-Chaverri, Francisco León, Claudia González-Espinosa, Manuel Jiménez-Estrada, Ricardo Reyes-Chilpa, Jorge Ivan Castillo-Arellano

**Affiliations:** 1 Instituto de Química, Universidad Nacional Autónoma de México, Ciudad Universitaria, Mexico City 04510, Mexico; 2 Posgrado en Producción Animal, Departamento de Zootecnia, Universidad Autónoma Chapingo, Chapingo, Texcoco 56230, Mexico; 3 Facultad de Química, Departamento de Biología, Universidad Nacional Autónoma de México, Mexico City 04510, Mexico; omarnoel@quimica.unam.mx (O.N.M.-C.); pedraza@unam.mx (J.P.-C.); 4 Department of Drug Discovery and Biomedical Sciences, College of Pharmacy, University of South Carolina, Columbia, SC 29208, USA; jleon@mailbox.sc.edu; 5 Departamento de Farmacobiología y Centro de Investigación Sobre el Envejecimiento, Centro de Investigación y de Estudios Avanzados del Instituto Politécnico Nacional, Sede Sur, Tlalpan, Mexico City 14330, Mexico; cgonzal@cinvestav.mx; 6 Laboratorio de Reprogramación Celular, Instituto Nacional de Neurología y Neurocirugía Manuel Velasco Suárez, Mexico City 14269, Mexico

**Keywords:** xanthone V, anti-allergy, jacareubin derivatives, methylation, acetylation, anti-inflammatory

## Abstract

Jacareubin (**2**), nujiangexanthone A, and α-mangostin display the highest anti-allergic effects among the active xantones through still not well-known mechanisms. This study investigates the SAR of jacareubin, its precursor xanthone V (**1**) and their peracetylated (**1a** and **2a**), permethylated (**1b** and **2b**) derivatives and their anti-allergic and anti-inflammatory effects. To characterize the inhibitory effect of jacareubin, **2a** and **2b** on the anaphylactic reaction, we first utilized in vitro models of bone marrow derived mast cells (BMMCs), determining their capacity of inhibiting the IgE/Antigen-induced degranulation, myeloperoxidase (MPO), and xanthine oxidase (XO) activation. Also, we utilized in vivo models of passive cutaneous anaphylaxis (PCA) and TPA-induced ear edema. In vitro tests showed that the compound **2b** was more effective than jacareubin in the inhibition of BMMCs degranulation. Besides, in vivo models of PCA revealed that the fourth cyclized ring of jacareubin is the critical structural element for anti-allergic efficacy, as compound **1** was less effective. Additionally, hydroxyl groups were found to be essential for inhibiting MPO. Jacareubin was the only tested xanthone that directly inhibited XO, a result supported by molecular docking. Overall, jacareubin represents a promising multi-target scaffold that could be used for developing new treatments for inflammatory and allergic diseases.

## 1. Introduction

**Figure 1:**
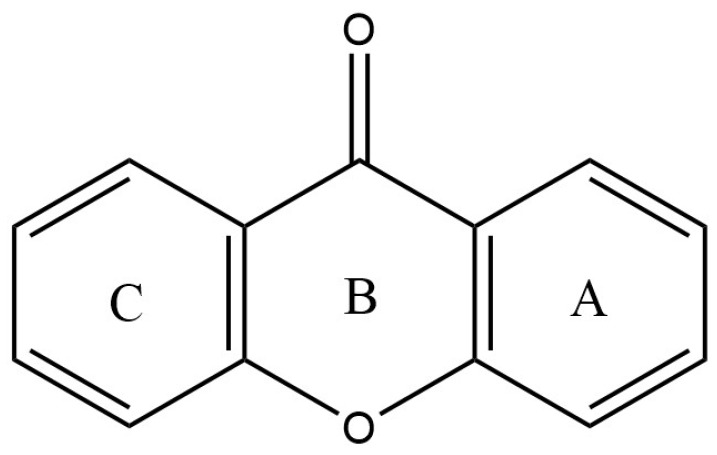
Xanthones are formed by two benzene rings (A, C) fused with a central γ-pyrone group (B).

Xanthones are compounds that exhibit structural diversity and exert many biological effects [1]. They are found in higher plant families such as Gentianaceae, Clusiaceae, Callophylaceae, and Moraceae, as well as in fungi and lichens [2]. Xanthones are formed from dibenzo-γ-pyrone, which is γ-pyrone condensed with two benzene rings from hybrid shikimate and acetate pathways (Figure 1) [1]. They are classified into several structural groups, including: (A) simple oxygenated xanthone, for example, 2-hydroxyxanthone; (B) xanthone glycosides, such as mangiferin, characterized by the presence of sugar moiety attached through C-C or C-O bonds; (C) prenylated xanthones, including allanxanthone-A; (D) bisxanthones (dimeric structures) such as ploiarixanthone; and (E) xanthonolignoids such as cadensin C [3].

Several xanthones with therapeutic potential have been identified [1], and around 44 xanthones have shown anti-inflammatory potential, being evaluated mainly in in vitro models using phagocytic cells such as the macrophage cell line RAW264.7, neutrophils, the fibroblast 3T3-L1 cell line and synovial cells. Also, in vivo models have been used, such as a bacterial lipopolysaccharide (LPS) or carrageenan-induced inflammation in rats or the ovalbumin-induced allergic asthma in mice [4]. Those experiments have identified several mechanism of action behind to the xanthone’s anti-inflammatory effects: (A) suppression NF-κB activity (mangiferin and α-mangostin); (B) inhibition of ERK1/2, JNK, and p38-dependent pathway activation (6′-O-acetyl mangiferin); (C) direct inhibition of the spleen tyrosine kinase (Syk), which prevents the downstream signaling required for mast cell degranulation (nujiangexanthone A); (D) antioxidant and cytoprotective effects by activating the Nrf2 pathway, which induces the expression of heme oxygenase-1 (HO-1) and other antioxidant enzymes like SOD and catalase (mangiferin); (E) inhibition of IgE/Ag-induced Ca^2+^ mobilization needed for mast cell activation (jacareubin, α- and γ-mangostins) [4,5,6,7,8,9,10,11].

Some xanthones have demonstrated efficacy in treating IgE-mediated allergic disorders, such as asthma, with compounds like mangiferin and mangostins attenuating ovalbumin (OVA)-induced airway inflammation [10,12,13,14,15]. They reduce eosinophil infiltration, decrease airway hyper-responsiveness (AHR), and rebalance the Th1/Th2 cytokine ratio [14,16,17]. Mangiferin, in particular, suppresses Th9 and Th17 responses while promoting an increase in regulatory T (Treg) cell populations [18]. Additionally, jacareubin, isolated from *Calophyllum brasiliense*, is among the most potent inhibitors of mast cell degranulation (IC_50_ = 46 nM). Its mechanism of action involves blocking FcεRI-induced extracellular calcium influx, reducing reactive oxygen species (ROS) production, and inhibiting xanthine oxidase activity. Jacareubin also exhibited strong inhibitory effects on passive cutaneous anaphylaxis (PCA) in mice, achieving 74–84% inhibition at doses of 11–33 mg/kg [6]. Notably, this compound did not show toxicity in immune cells (up to 50 µM) or mice (up to 250 mg/kg) [19].

Previously, we identified more than 100 natural compounds with anti-allergic activity where jacareubin, nujiangexanthone A, and α-mangostin were the most active xanthones [20]. Based on these findings, jacareubin was identified as a promising scaffold for the development of new derivatives with therapeutic applications. Jacareubin exhibited anti-inflammatory and anti-allergic activity in vitro using a mast cell model and in vivo using a PCA model in mice. However, the structure-activity relationship of jacareubin as an inhibitor of xanthine oxidase remains unexplored. In this study, we evaluated jacareubin, its natural precursor xanthone V, and their methylated and acetylated derivatives to assess the contribution of hydroxyl groups to the biological activity of the compound. The effect of jacareubin substituents were investigated using in vitro models of BMMC, analyzing degranulation, myeloperoxidase and xanthine oxidase activity. Also, we utilized in vivo models of PCA and TPA-induced ear edema.

## 2. Results

### 2.1. Chemical Modification of Jacareubin and Xanthone V

**Figure 2:**
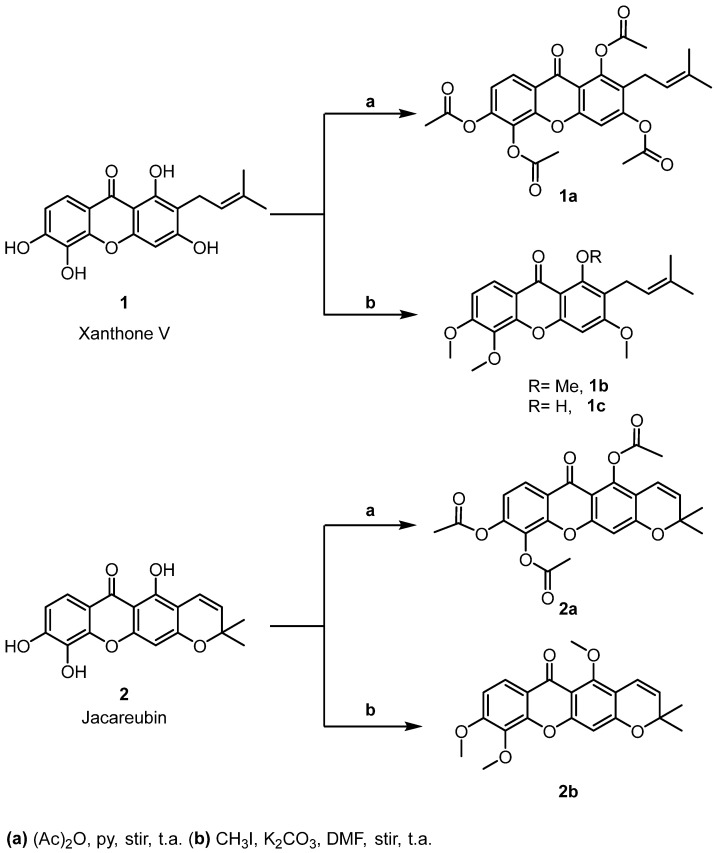
Structures of natural xanthones jacareubin, xanthone V, and their derivatives.

The acetylation and methylation reactions of compounds **1** and **2** (Figure 2) yielded good results (37–97%) and a purity of 90–97% as determined by High Performance Liquid Chromatography HPLC (Supplementary Figure S8). Upon acetylation, both compounds **1** and **2** produced their corresponding peracetylated derivatives, **1a** and **2a**. Excess methyl iodide treatment of compound **1** afforded both the permethylated derivative **1b** and the trimethylated product **1c**. In contrast, compound **2** yielded only the permethylated derivative **2b** under the same conditions. Chemical modification of compound **2** through methylation of the hydroxyl substituents resulted in a decrease in the polarity and melting point of the new molecule to between 74–76 °C. This difference in properties may be associated with the loss of hydrogen bonds formed by the interaction of the hydroxyl substituents with the carbonyl group of other jacareubin molecules in the crystal formed, as observed in X-ray diffraction [6].

### 2.2. Effect of Xanthone Methylation and Acetylation on FcεRI-Induced BMMC Degranulation

**Figure 3:**
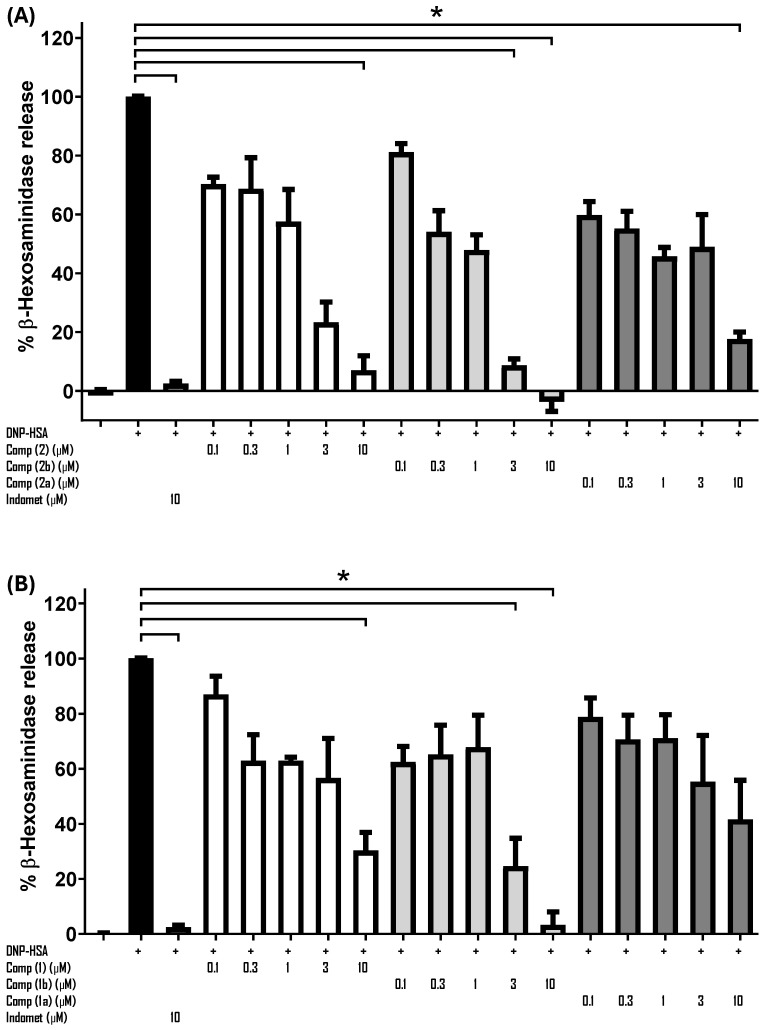
Inhibition of anaphylactic degranulation in IgE-sensitized BMMCs. (**A**) Effect of compound **2**, **2a** and **2b**; (**B**) Effect of compound **1**, **1a** and **1b**. Kruskal Wallis test (* *p* < 0.05%, n = 3, SEM).

The effect of the methylated and acetylated derivatives of compound **2** on IgE/Ag-induced mast cell degranulation was tested utilizing IgE-sensitized BMMCs and determining the amount of β-hexosaminidase release to the cell supernatant (Figure 3). As observed, compound **2b** showed better inhibitory capacity than compound **2**, an effect that was clearly detected when 3 μM of either compound was used. Acetylated compound of jacareubin **2a** showed weak capacity of inhibition of IgE/Ag-induced degranulation, since significant effect was observed only at 10 μM (Figure 3, Panel A).

On the other hand, compound **1** showed lower inhibitory capacity compared to compound **2** (Figure 3, Panel B). Although at 3 μM, compound **1** and **2** show similar inhibitory capacity, the dose-response effect was different. Only 10 μM compound **1** was able to fully inhibit FcεRI-induced degranulation. On the other hand, compound **1a** did not show any significant inhibitory capacity at lower concentrations, but diminished IgE/Ag-induced degranulation at 10 μM (Figure 3, Panel B). We included indomethacin as a positive control.

These results indicated that the absence of hydroxyl groups due to methylation did not affect the inhibitory capacity of compounds **1** and **2** in BMMCs. On the contrary, they could potentiate that capacity. To investigate if the inhibitory effects of the jacareubin derivatives could be observed in vivo, we decided to evaluate their anti-allergic activity in the murine PCA model.

### 2.3. Methylation of Compound ***2*** Does Not Affect Its Anti-Allergic Activity In Vivo

**Figure 4:**
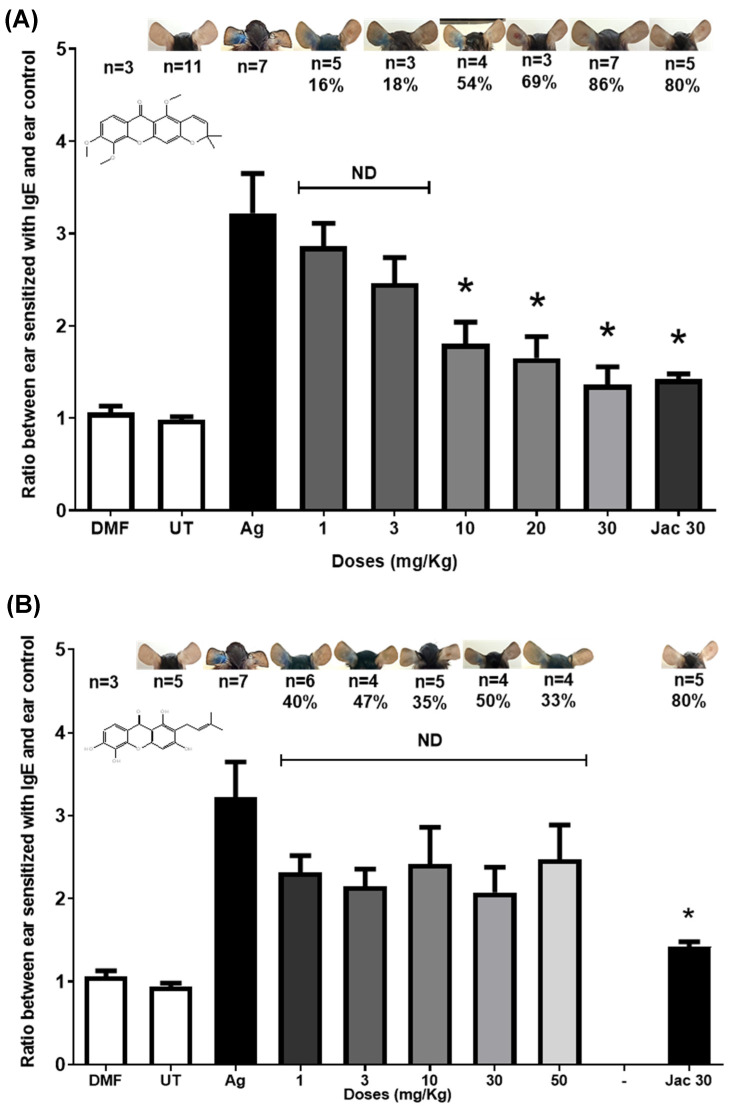
Inhibition of passive cutaneous anaphylaxis in mice by xanthones. (**A**) compound **2b**; (**B**) compound **1**. UT = untreated; Ag = antigen; compound **2** (Jac 30 mg/kg) was included as a control; DMF = Dimethylformamide as a blank. ANOVA one way and Post hoc Dunnet test (* *p* < 0.05%, n = 3–7, SEM).

The capacity of compound **2b** to inhibit the PCA reaction in mice was similar to compound **2** (Figure 4, Panel A), while compound **1** which does not have a cyclized fourth ring and possesses a fourth hydroxyl substituent, showed a weak inhibition of the allergic response even at doses of 50 mg/kg (33%) (Figure 4, Panel B). This suggests that the fourth ring of the molecule is an important structural element of the anti-allergic activity, above solubility and the number of hydroxyls, as we had previously suggested [6]. We included compound **2** (30 mg/kg) as a positive control. On the other hand, compound **1b**, which is a derivative of xanthone V, was unable to halve the anaphylactic response in mice at a dose of 10 mg/kg (Figure S9, Panel A). Because the acetylated derivatives of compounds **1** and **2** showed the weakest inhibitory responses to the allergic reaction, they were not considered for evaluation of the anti-inflammatory activity using phagocytes, neither for the inhibition of the MPO and xanthine oxidase enzymes, which are presented below. We included loratadine as a positive control (Figure S9, Panel B).

### 2.4. Inhibition of TPA-Induced Inflammation with Xanthones

**Figure 5:**
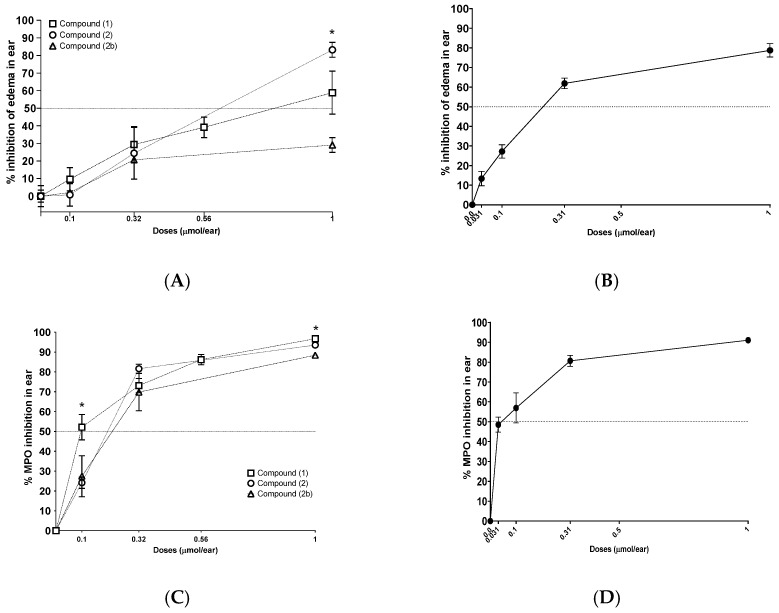
TPA-induced inflammation test in the mouse ear treated with xanthones. (**A**) Edema inhibition test for compounds **1**, **2**, and **2b**; (**B**) Edema inhibition test for positive control indomethacin; (**C**) MPO inhibition test for compounds **1**, **2**, and **2b**; (**D**) MPO inhibition test for positive control indomethacin. Kruskal Wallis test (* *p* < 0.05%, n = 5, SEM).

The classic acute allergic response is mediated by mast cells, and the subsequent inflammatory response is mediated by other cells such as neutrophils and macrophages. Three compounds **1**, **2**, and **2b**, were assessed using the TPA-induced mouse ear inflammation test. Significant differences were observed only at a concentration of 1 μmol/ear between compound **2** (ED_50_ = 0.54 ± 0.06 μmol/ear), and compound **2b** (ED_50_ = 1.46 ± 0.06 μmol/ear), but no differences were observed between compound **1** and **2** (ED_50_ = 0.71 ± 0.05 μmol/ear) (Figure 5, Panel A). In the case of the inhibition of myeloperoxidase (MPO) activity in mouse ear tissue, significant differences were observed at a dose of 0.1 μmol/ear between compound **1** (IC_50_ = 0.09 ± 0.03 μmol/ear) and compound **2** (IC_50_ = 0.15 ± 0.04 μmol/ear), but no difference was observed between compound **2** and **2b** (IC_50_ = 0.19 ± 0.13 μmol/ear). Also, the three compounds showed significant differences in inhibition at 1 μmol/ear (Figure 5, Panel C). This suggests that the hydroxyl groups in compounds **1** and **2** play a crucial role in MPO inhibition. We included indomethacin as a positive control (Figure 5, Panel B and D).

**Figure 6:**
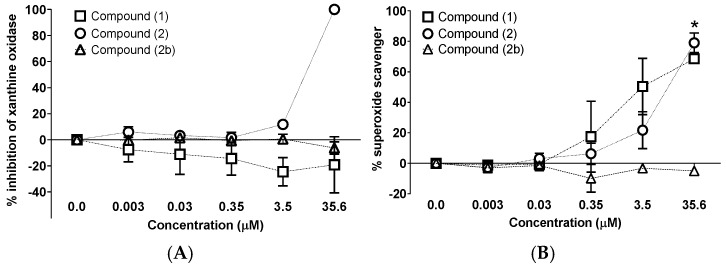
Inhibition of xanthine oxidase by compound **1**, **2**, and **2b**. (**A**) Inhibition of superoxide production by the xanthine oxidase; (**B**) Inhibition of uric acid product by xanthine oxidase. Kruskal Wallis test (* *p* < 0.05%, n = 3, SEM).

Compound **2** was the only one of the two natural xanthones that inhibited xanthine oxidase (XO) activity (IC_50_ = 5.61 ± 2.18 µM). Compound **1** and **2b**, on the other hand, showed no effect even at the highest concentration (35.6 µM) that was evaluated (Figure 6, Panel A). However, the production of superoxide radical (generated by the same enzyme) was significantly reduced by the natural xanthones compound **2** (IC_50_ = 10.89 ± 0.1 µM) and compound **1** (IC_50_ = 5.46 ± 0.27 µM). Compound **2b** did not inhibit xanthine oxidase in any of the two assays (Figure 6, Panel B). We included allopurinol as a positive control (Figure S10).

### 2.5. Molecular Docking Analysis Confirms In Vitro and In Vivo Results

**Figure 7:**
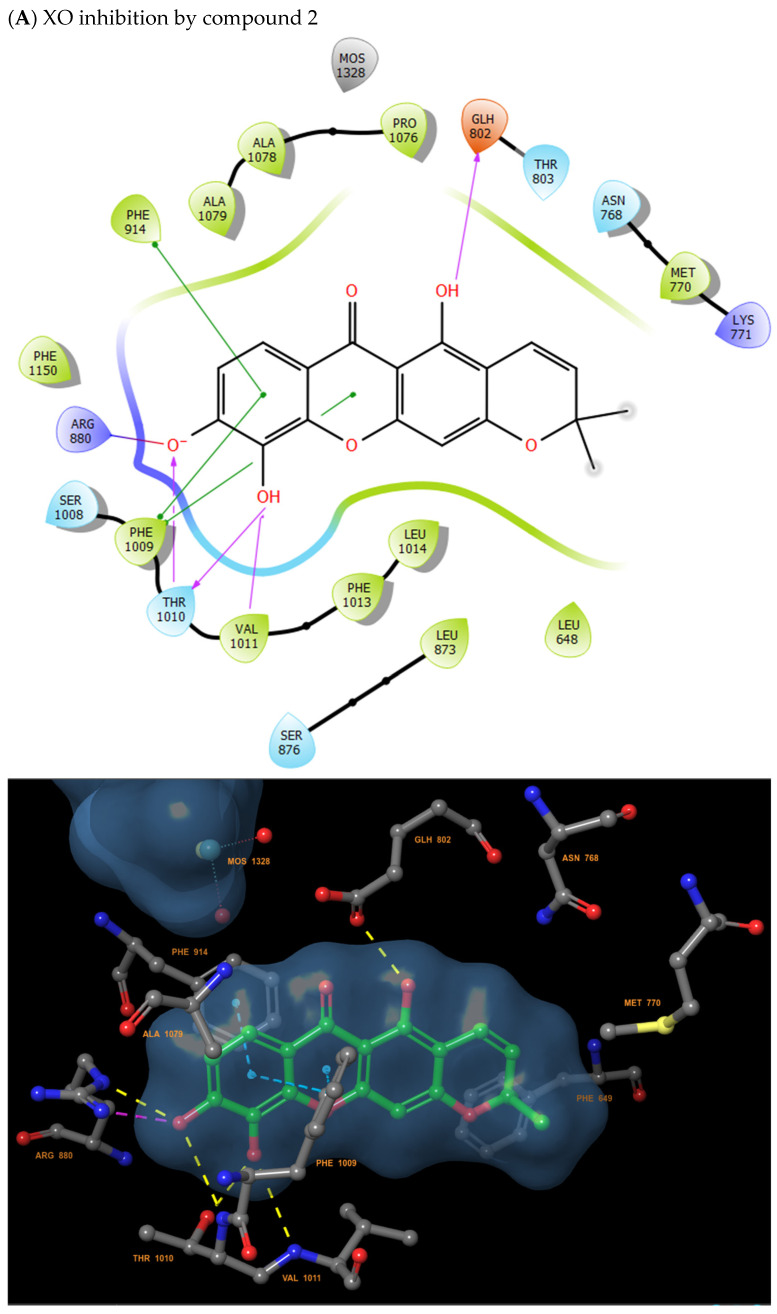

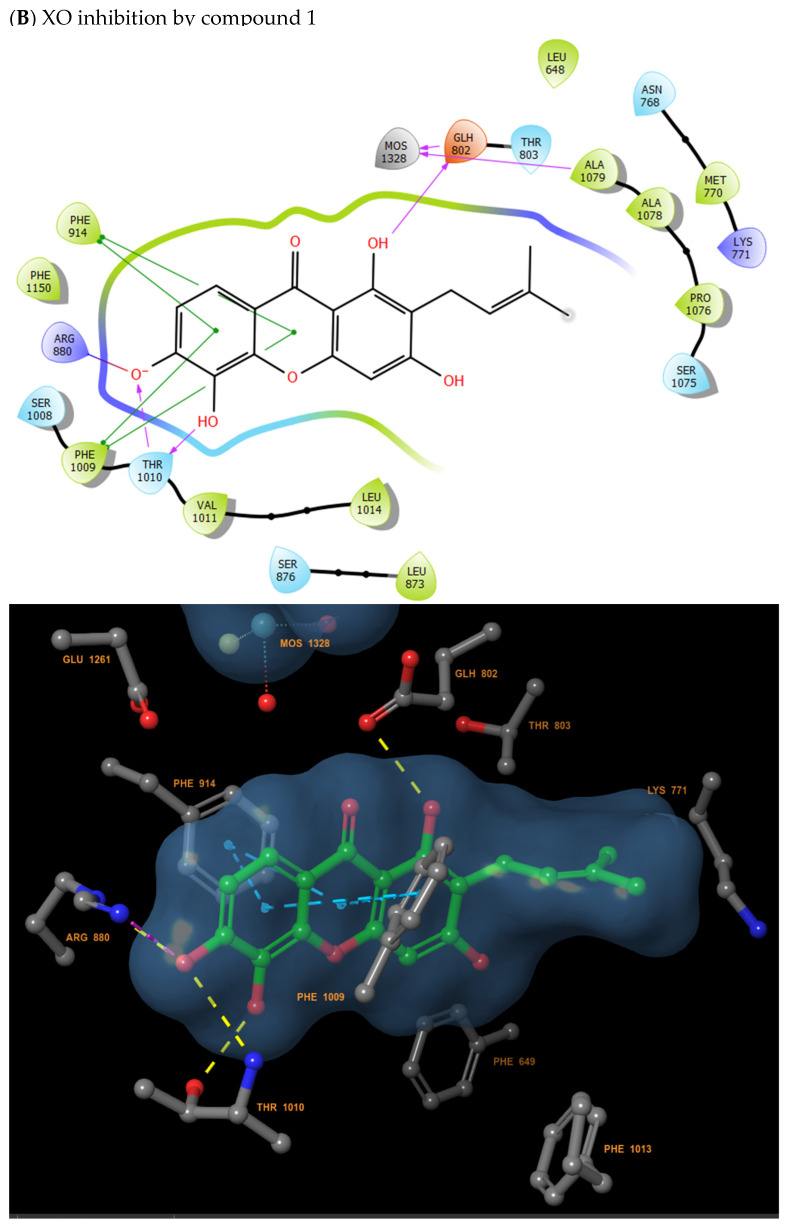
Docking of xanthine oxidase. (**A**) inhibition XO by compound **2**; (**B**) inhibition XO by compound **1**.

**Figure 8:**
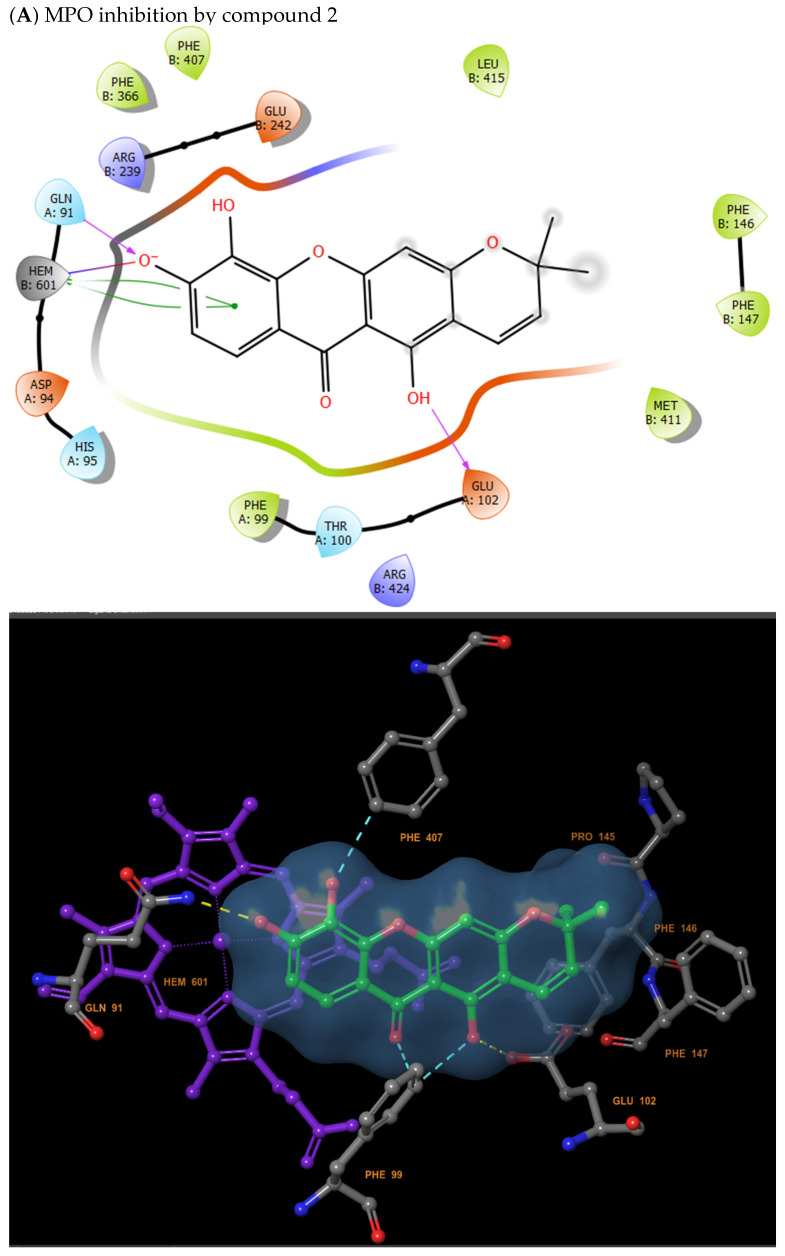

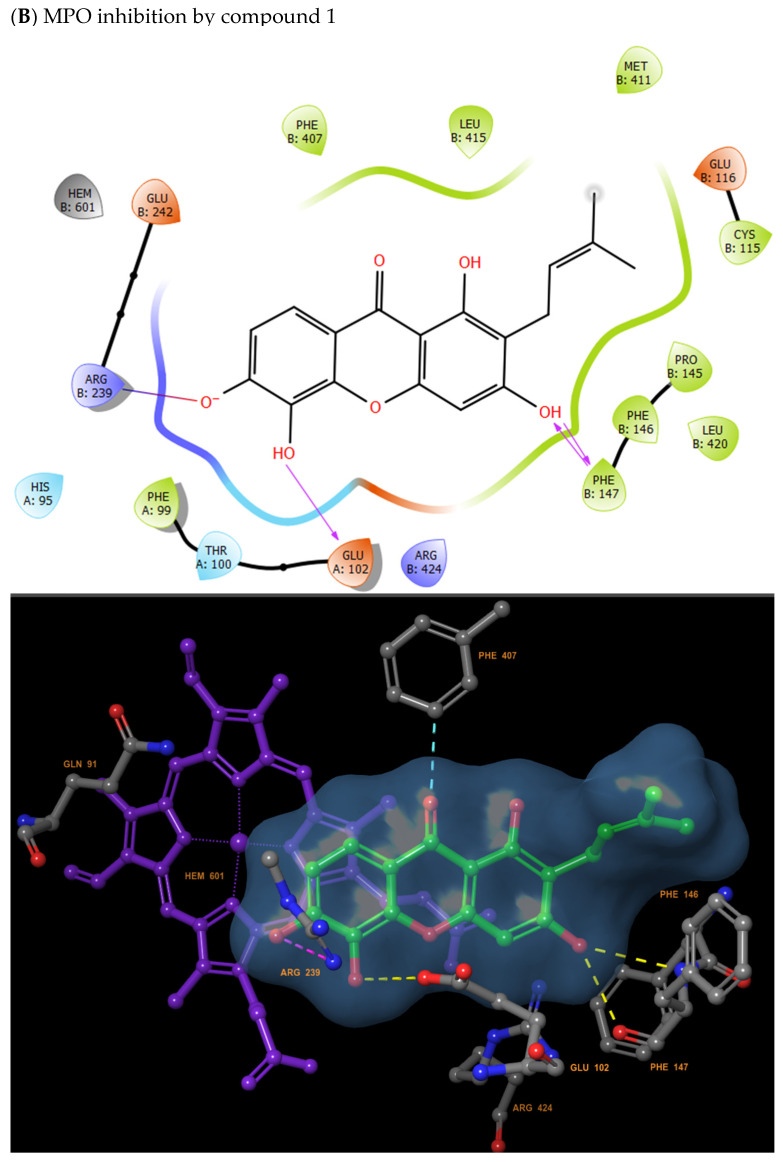
Docking of MPO. (**A**) inhibition MPO by compound **2**; (**B**) inhibition MPO by compound **1**.

To better explain how the inhibition of MPO and xanthine oxidase enzymes can occur, an in silico analysis using the Schrödinger software for molecular docking of compounds **1** and **2** was performed. Results of in silico analysis showed similar results to those observed in the in vitro results. Compound **2** exhibited greater inhibitory capacity against xanthine oxidase (Table 1), with a strong hydroxyl group binding to ARG880 and multiple hydrogen bonds to GLH802, THR1010 and VAL1011 (Figure 7). On the other hand, compound **1** showed greater inhibitory capacity against MPO (Table 2), with a strong hydroxyl group binding to ARG239 and multiple hydrogen bonds to GLU102, and PHE147 (Figure 8).

## 3. Discussion

It has been suggested that compound **1** is the precursor of compound **2** [21] since compound **1** does not have a cyclized fourth ring and only requires 2H to cyclize (Figure 2). Furthermore, both compounds **1** and **2** are found combined in the acetone and methanolic extracts of *Calophyllum brasiliense* heartwood. The xanthone skeleton has been described with good hydrolytic and thermal oxidative stability and is, therefore, considered a raw material for the development of polymeric and medicinal structures [3,22]. A three-step method for synthesizing a xanthone similar to compound **2** has recently been described [1], which could be used to synthesize compound **2** for industrial applications.

Compound **1** is a molecule with high polarity, determined by its four hydroxyl substituents, and presents high solubility in ethanol (60 mM), while compound **2** presents a solubility of 30 mM in ethanol. Furthermore, when compound **2** was transformed into compound **2b**, its polarity decreased substantially to 10 mM in DMSO, as it was no longer soluble in ethanol. The antioxidant capacity of xanthones has been described as depending on the presence of hydroxyl substituents, with positions 1, 3, and 6 being the most favorable antioxidant activity [1].

We had previously suggested that the anti-allergic activity of compound **2** depends primarily on the three hydroxyl substituents [6]. However, the results obtained in the PCA model demonstrated that methylation of the hydroxyl substituents does not diminish compound **2** anti-allergic activity in vivo when administered (Figure 4, Panel A). Considering these new results, we suggest that the fourth ring of the compound **2** molecule is responsible for the inhibitory activity on the allergic response. This is based on the result showing that compound **1** also failed to inhibit the allergic response in the PCA at low doses, nor could it surpass the inhibitory activity of compound **2** in terms of the percentage of BMMC degranulation (Figure 3).

It is worth mentioning that the methylation of compounds **1** and **2** had exerted a positive effect, improving the inhibition of BMMC degranulation in vitro. Acetylation, on the other hand, produced the opposite effect, suggesting that this modification profoundly affects the solubility of the derivatives of the two natural products and its interaction with molecular targets (Figure 3). However, compound **1b** in vivo could not inhibit anaphylactic reaction at 10 mg/kg.

Part of the anti-inflammatory activity of xanthone derivatives has been determined by their ability to scavenge the superoxide anion and elastase secretion in human neutrophils stimulated with fMLP/CB. This model is like TPA model, as it activates the same signaling pathway. A compound jacareubin-like xanthone known as 5,9-Dihydroxy-2,2-dimethylpyrano[3,2-b]xanthen-6(2H) showed an IC_50_ = 0.46 μg/mL against superoxide [23,24]. Compound **2**, on the other hand, showed an IC_50_ of 10.88 μM (3.54 μg/mL) for the inhibition of superoxide production by xanthine oxidase.

Mangiferin aglycone derivatives have been described with activity against xanthine oxidase, with IC_50_ values ranging from 4.67 ± 0.35 μM to 21.73 ± 1.52 μM for the mangiferin aglycone (norathyriol). These results show that the IC_50_ of compound **2** against xanthine oxidase falls within these parameters. Considering that allopurinol, the reference drug, has an IC_50_ of 24.40 ± 0.5 μM [25]. When comparing the molecular docking of one of the norathyriol derivatives with compound **2**, no similarities were found in the interacting amino acids of both molecules, possibly because a different crystal structure of the enzyme was used in the PDB.

## 4. Materials and Methods

### 4.1. Reagents

The reagents used for the experiments 12-O-tetradecanoylphorbol-13-acetate (TPA), 3,3′,5,5′,tetramethyl-benzidine (TMB), Anti-dinitrophenyl monoclonal immunoglobulin type E (IgE), bovine serum albumin (BSA), deoxyribonucleic acid (single stranded from salmon testes), dihydroethidium (DHE), dimethyl sulfoxide (DMSO), dinitrophenyl-human serum albumin (DNP-HSA), diphenyleneiodonium chloride (DPI), Ethylenediaminetetraacetic acid (EDTA), egtazic acid (EGTA), Evans blue dye, hydrogen peroxide (H_2_O_2_), HEPES, hexadecyltrimethyl-ammonium bromide (HTAB), hexadecyltrimethylammonium bromide (HTAB), indomethacin, N,N-dimethylformamide (DMF), sodium carbonate (Na_2_CO_3_), nicotinamide adenine dinucleotide (NADPH), nitrotetrazolium blue (NBT), phenylmethanesulfonyl fluoride (PMSF), phosphate-buffered saline (PBS), p-nitrophenyl-N-acetyl-β-D glucosaminide (P-NAG), sodium Fluoride (NaF), triton X-100, Trizma base HCl, xanthine oxidase and xanthine, were purchased to Sigma-Aldrich (St. Louis, MO, USA). Organic solvents were purchased with J. T. Baker (Center Valley, PA, USA). Ketamine and xilazine were purchased with PISA agropecuaria (Guadalajara, Jalisco, Mexico). The obtention of xanthone V (**1**), tetraacethylxanthone V (**1a**), tetramethylxanthone V (**1b**), jacareubin (**2**), triacethyljacareubin (**2a**), and trimethyljacareubin (**2b**) is described below.

### 4.2. Equipment for Analytical Determinations

Melting points were measured using a Fisher Johns Apparatus (Scorpion Scientific; Chicago, IL, USA), and values are uncorrected. Mono- and bi-dimensional NMR spectra were completed using a Bruker AVANCE III HD 500 MHz (Cambridge Isotope Laboratories, Tewksbury, MA, USA) using DMSO-d_6_, acetone-d_6_, and CDCl_3_ as solvents and references. Spectra were processed using MestReNova software 14.2.0. The mass spectra were acquired on an AccuTOF JMS-T100LC (Tokyo, Japan). Analyses were performed in positive ion mode.

### 4.3. Collection of Plant Material and Isolation of Compounds ***1*** and ***2***

The predominant xanthones in the heartwood of *Calophyllum brasiliense* are compounds **1** and **2**. Isolation was carried out protocol as follows: 711.5 g of heartwood shavings were extracted at room temperature using hexane, ethyl acetate, acetone and methanol, and concentrated in a vacuum rotary evaporation apparatus [6]. Part of the dry extract 74 g was subjected to column chromatography Silica gel 60 (Merck; Darmstadt, Germany) eluting with hexane-ethyl acetate (7:3). Monitoring was performed with TLC at 366 nm and visualized with 1% ceric sulfate in 1 N sulfuric acid.

A mixture (9.85 g) of three xanthones, identified as jacareubin (xanthone III), xanthone IV, and xanthone V from fractions 138–227. A portion of this mixture (6 g) was purified by column chromatography (1 m height, 5 cm diameter, silica gel 70/230, 120 g), eluting with hexane and then gradually increasing polarity with ethyl acetate. Fractions eluted with a mixture 87:13 yielded 1 g of 99% pure compound **2** [JIC2.1] after recrystallization from acetone.

Further fractions [JIC3.1] (5 g) underwent column chromatography (40 cm height, 3 cm diameter) using 50 g of C18 Silica gel (Macherey-Nagel; Düren, Germany). Compound **1** was purified using a mobile phase of acetonitrile:water (7:3) with 0.5% acetic acid. Fifteen subfractions were obtained and analyzed with thin-layer chromatography (TLC) on G/UV254 plates, visualized under UV light (254 and 366 nm) and by spraying with 1% ceric sulfate in 1 N sulfuric acid. Subfractions containing compound **1** were pooled (2 g).

Xanthone V (**1**). 1,3,5,6-tetrahydroxy-2-(3,3-dimethylbut-2-en-1-yl)-9H-xanthen-9-one was obtained as a light-yellow powder, melting point 240–242 °C. ^1^H NMR (500 MHz, DMSO-d_6_). δ 13.39 (1H, s, 1″-OH), 10.90 (1H, s,b, 4″-OH), 7.48 (1H, d, 8-H), 6.89 (1H, d, 7-H), 6.49 (1H, s, 4-H), 5.18 (1H, m, 2′-H), 3.21 (2H, d, 1′-CH_2_), 1.73 (3H, s, 5′-CH_3_), 1.62 (3H, s, 4′-CH_3_) and ^13^C NMR (125 MHz, DMSO-d_6_) δ 180.17 (C-9), 163.28 (C-3), 160.20 (C-1), 155.53 (C-4a), 152.21 (C-6), 146.44 (C-5a), 132.84 (C-5), 131.02 (C-3′), 122.81 (C-2′), 116.35 (C-8), 113.47 (C-8a), 113.37 (C-7), 110.15 (C-2), 101.64 (C-9a), 93.72 (C-4), 25.96 (C-4′), 21.40 (C-1′) and 18.17 (C-5′) (Figure S1). Mass spectra (DART+) *m*/*z* (%): 329 M^+^ + 1 (97); 273 (100).

Jacareubin (**2**). 5,9,10-trihydroxy-2,2-dimethyl-2H,6H-pyrano[3,2-b]xanthen-6-one was obtained as yellow cubic crystalline prisms 1–5 mm long, melting point 260–270 °C. ^1^H NMR (500 MHz, (CD_3_OD). δ 7.59 (1H, d, 7-H), 6.88 (1H, d, 1′-H), 6.68 (1H, d, 8-H), 6.42 (1H, s, 4-H), 5.67 (1H, d, 2′-H), 1.48 (6H, s, 4′-CH3, 5′-CH3) and ^13^C NMR (125 MHz, (CD_3_OD) δ 180.56 (C-9), 160.22 (C-1), 157.30 (C-3), 157.06 (C-4a), 151.82 (C-6), 146.13 (C-5a), 132.31 (C-5), 127.34 (C-2′), 116.12 (C-8), 114.77 (C-7), 113.43 (C-8a), 112.50 (C-1′), 104.14 (C-2), 102.36 (C-9a), 94.58 (C-4), 77.87 (C-3′) and 27.19 (C-4′ and C-5′) (Figure S2). Mass spectra (DART+) *m*/*z* (%): 327 [M^+^ + 1] (100); 257 (23), using ionization mode Direct Analysis in Real Time.

### 4.4. General Procedure for the Synthesis of Acetylated Derivatives of Compounds ***1*** and ***2***

In a round-bottom flask, the precursors, compound **1** (160.2 mmol) or compound **2** (0.148 mmol) dissolved in pyridine (1–2 mL) were added dropwise acetic anhydride (2–3 mL). The reaction mixture was stirred at room temperature for 24 h. The solution was then poured onto iced water (10 mL) and extracted with ethyl acetate. The combined organic extracts were washed successively with 1N HCl, NaHCO_3_, and brine. The organic phase was then dried over anhydrous Na_2_SO_4_, filtered, and concentrated under reduced pressure, and the compound **1a** (Yield = 63.5 mg, 97%) and **2a** (Yield = 74 mg, 91%) were formed.

(**1a**). 2-(3-methylbut-2-en-1-yl)-9-oxo-9H-xanthene-1,3,5,6-tetrayl tetraacetate was obtained as a light-yellow powder with a melting point of 240–242 °C. ^1^H NMR (500 MHz, CDCl_3_). δ (ppm) 8.15 (1H, s), 7.27 (1H, s), 7.21 (1H, d), 5.03 (1H, tt), 3.31 (2H, s), 2.51 (3H, s), 2.46 (3H, s), 2.37 (6H, s) 1.78 (3H, s), 1.70 (3H, s) and ^13^C NMR (125 MHz, CDCl_3_) δ (ppm) 174.09 (C-7), 169.18 (C-35), 168.06 (C-40), 167.48 (C-47), 167.18 (C-51), 155.11 (C-9), 153.83 (C-13), 149.03 (C-11), 148.65 (C-5), 147.42 (C-3), 132.82 (C-19), 130.82 (C-4 and C-12), 124.60 (C-1), 120.91 (C-16), 118.85 (C-6), 112.82 (C-2 and C-8), 109.84 (C-14) 25.63 (C-20), 23.48 (C-15), 21.12 (C-46 and C-41), 20.72 (C-54), 20.29 (C-39) and 17.95 (C-25) (Figure S3). Mass spectrometry *m*/*z*: 497 [M + 1]^+^ (100).

(**2a**). 2,2-dimethyl-6-oxo-2H,6H-pyrano[3,2-b]xanthene-5,9,10-triyl triacetate was isolated as a light-yellow powder, with a melting point between 260–270 °C. ^1^H NMR (500 MHz, CDCl_3_). δ 8.12 (1H, d), 7.17 (1H, d), 6.74 (1H, s), 6.50 (1H, d), 5.77 (1H, d), 2.53 (3H, s), 2.45 (3H, s), 2.37 (3H, s), 1.51 (6H, s) y ^13^C NMR (125 MHz, CDCl_3_) δ 173.81 (C-7), 169.23 (C-33), 167.59 (C-22), 167.23 (38) 159.14 (C-13), 157.55 (C-9), 148.43 (C-5), 146.99 (C-3), 145.57 (C-11), 130.77 (C-16), 124.05 (C-1), 121.02 (C-2 y C-4), 118.55 (C-6), 114.98 (C-15), 112.50 (C-8), 108.89 (C-12), 102.39 (C-14), 78.50 (C-17), 28.54 (C-42 y C-43), 21.08 (C-41), 20.71 (23) and 20.24 (C-32) (Figure S6). Mass spectrometry was performed using the DART+ technique, finding a molecular ion *m*/*z* (%): 453 [M + H]^+^ (100). From the acetylation reaction procedure of compound **2**, two mixed products were obtained, which were separated by three times washing with MeOH, and recovered only compound **2a**.

### 4.5. General Procedure for the Synthesis of Methylated Derivatives of Compounds ***1*** and ***2***

Briefly, compound **1** (0.154 mmol, 4 eq) or **2** (0.153 mmol, 3 eq) were dissolved in DMF (5 mL). After 15 min, iodomethane (86 μL, 9eq and 76 μL, 8 eq) was added to the flask, and the resulting mixture was stirred overnight. K_2_CO_3_ (4 eq) was added, and the reaction was stirred for 65–106 h. The reaction stopped slowly and was neutralized with 0.1 M HCl. Subsequently dried under vacuum and were purified by column chromatography. Compounds **1b** (Yield = 43.7 mg, 37%), and 1c (Yield = 1.2 mg, ~1%) were obtained from compound **1**. For compound **2** was formed only compound **2b** (Yield = 49.9 mg, 88%).

(**1b**) 1,3,5,6-tetramethoxy-2-(3-methylbut-2-en-1-yl)-9H-xanthen-9-one was isolated as a white powder with a melting point between 240–242 °C. ^1^H NMR (500 MHz, CDCl_3_). δ 8.03 (1H, d), 6.95 (1H, d), 6.80 (1H, s), 5.17 (1H, tt), 4.01 (3H, s), 3.99 (3H, s), 3.95 (3H, s), 3.91 (3H, s), 3.40 (2H, d), 1.80 (3H, s), 1.67 (3H, s) and ^13^C NMR (125 MHz, CDCl_3_) δ 174.58 (C-11), 162.99 (C-17), 158.73 (C-15), 157.72 (C-5), 156.67 (C-13), 149.47 (C-3), 135.83 (C-4), 131.66 (C-23), 122.58 (C-20), 122.26 (C-16), 121.21 (C-1), 117.81 (C-2), 109.93 (C-12), 108.28 (C-6), 95.59 (C-18), 62.20 (C-31), 61.57 (C-8), 56.34 (C-35), 56.03 (C-9), 25.75 (C-29), 22.31 (C-19), and 17.83 (C-24) (Figure S5). Mass spectrometry was performed by DART+, obtaining a molecular ion of *m*/*z* (%): 385 [M^+^ + 1] (100).

(**1c**). 1-hydroxy-3,5,6-trimethoxy-2-(3-methylbut-2-en-1-yl)-9H-xanthen-9-one was isolated as a white powder with a melting point between 240–242 °C. ^1^H NMR (500 MHz, CDCl_3_). δ 12.97 (1H, s, 1″-OH), 7.99 (1H, d), 6.97 (1H, d), 6.54 (1H, s), 5.23 (1H, tt), 4.01 (6H, d), 3.94 (3H, s), 3.36 (2H, d) 1.80 (3H, s), 1.69 (3H, s) (Figure S4).

(**2b**). 5,9,10-trimethoxy-2,2-dimethyl-2H,6H-pyrano[3,2-b]xanthen-6-one) was obtained like a white powder with melting point 186–188 °C. ^1^H NMR (500 MHz, CDCl_3_). δ (ppm) 8.00 (1H, d, 8-H), 6.95 (1H, d, 1′-H), 6.74 (1H, d, 7-H), 6.72 (1H, s, 4-H), 5.68 (1H, d, 2′-H), 3.99 (3H, s, 6′-CH3), 3.98(3H, s, 8′-CH3), 3.94 (3H, s, 7′-CH3), 1.48 (6H, s, 4′-CH3, 5′-CH3) and ^13^C NMR (125 MHz, CDCl_3_) δ 174.68 (C-9), 159.06 (C-1), 158.59 (C-3), 156.84 (C-4a), 156.36 (C-6), 149.50 (C-5a), 135.88 (C-5), 130.16 (C-2′), 122.16 (C-8), 117.59 (C-9a), 116.08 (C-1′), 112.37 (C-8a), 110.13 (C-2), 108.32 (C-7), 101.01 (C-4), 77.88 (C-3′) 62.74 (C-6′), 61.57 (C-7′), 56.38 (C-8′) and 28.40 (C-4′ and C-5′) (Figure S7). Mass spectrometry was performed by DART+, obtaining a molecular ion of *m*/*z* (%): 369 [M^+^ + 1] (100).

The purity of compound **2** (99%) was determined by HPLC coupled with a UV–Vis Photodiode Array Detector and a Bruker Esquire 6000 Mass Spectrometer (Bruker; Billerica, MA, USA) [6]. Purity of compound **1** (90%) and **2b** (97%) was determined on an Agilent 1200 Series Binary SL system using a C-18 Eclipse Plus column (100 × 2.1 mm, 3.5 μm). The mobile phase was CH_3_CN/H_2_O (7:3) with 0.1% CH_3_COOH, utilizing both Waters 2996 UV–Vis and Bruker Esquire 6000 detectors, and showed retention times of 11.92 min and 16.72 min, respectively (Supplemental Figure S8).

### 4.6. Determination of β-Hexosaminidase Activity

Degranulation of bone marrow-derived mast cells (BMMCs) of C57BL6/J mice was assessed measuring β-hexosaminidase activity in cell supernatants, as described by Saito and Manetz [26,27]. Briefly, 2 × 10^6^ IgE-sensitized cells were harvested and resuspended in 1 mL of Tyrode’s/BSA buffer. Groups of cells in different vials were pre-treated for 15 min with 0.1% DMSO (vehicle) or various concentrations (0.1, 0.3, 1, 3, 10 μM) of compounds **1**, **1a**, **1b**, **2**, **2a**, and **2b**, followed by stimulation with 9 ng/mL DNP-HSA for 30 min at 37 °C. We included indomethacin (10 μM) as a positive control. After cooling on ice, samples were centrifuged (12,000 × g, 10 min, 4 °C). Supernatants (60 μL) were incubated with 1 mM P-NAG for 1 h at 37 °C. The reaction was halted with 120 μL of stop solution (0.1 M Na_2_CO_3_/0.1 M Na_2_HCO_3_), and β-hexosaminidase release was quantified at 405 nm in a plate reader (Tecan Sunrise; Männedorf, Switzerland). All procedures were approved by the CICUAL-Cinvestav Ethics Committee (CICUAL-0369-24).

### 4.7. Xanthine Oxidase Activity

The xanthine/xanthine oxidase reaction was used following the Pineda (2020) protocol to evaluate xanthine oxidase activity by measuring superoxide and uric acid production [28]. To assess the activity over xanthine oxidase of compounds **1**, **2**, and **2b**, 20 μL of these compounds at 0, 0.003, 0.03, 0.35, 3.5, and 35.6 μM were added to 150 μL of a solution containing 0.122 mM EDTA, 49 mM Na_2_CO_3_, 20 μM DHE, 0.122 mM xanthine, and 0.5 mg/mL salmon testes DNA [29]. DHE oxidation by superoxide generates ethidium, which intercalates with DNA; the resulting fluorescence was measured (Ex: 480 nm, Em: 610 nm) using a Synergy HT microplate reader (BioTek; Winooski, VT, USA).

An alternative assay for xanthine oxidase activity, based on uric acid formation, was conducted as follows: 20 μL of samples at various concentrations were added to 150 μL of a master mix containing 0.122 mM EDTA, 49 mM Na_2_CO_3_, 0.122 mM xanthine, and 30.6 μM NBT. Uric acid production (monitored at 295 nm) was initiated by adding 10 μL of 0.1 U/mL xanthine oxidase [28]. Compound **1**, **2**, and **2b** were resuspended in DMSO immediately before enzyme addition when necessary. The vehicle control alone did not affect enzyme activity. In parallel, an allopurinol curve was included as a positive control.

### 4.8. Passive Cutaneous Anaphylaxis Model (PCA)

Mice C57BL6/J were anesthetized via i.p. injection of 17 μL ketamine and 1.1 μL xylazine in saline (160 μL). For sensitization, 100 ng (5 μL) of monoclonal anti-DNP IgE was administered to the left ear, with saline in the right ear as a control. Twenty-four hours later, animals were administered i.p. 2 h prior with compounds **1** and **2b**, (1, 3, 10, 20, 30, or 50 mg/kg) or vehicle. After, 200 μg DNP-HSA in 1% Evans Blue dye via the tail vein and 30 min after, mice were euthanized; ear tissue was then weighed and incubated in DMF for 12 h at 65 °C [30,31]. Evans Blue extraction was quantified at 620 nm with a VersaMax spectrophotometer. All procedures were approved by the CICUAL-Cinvestav Ethics Committee (CICUAL-0384-24).

### 4.9. TPA-Induced Edema Model in the Mouse Ear

TPA-induced ear edema was evaluated in Swiss Webster mice (Charles River, Wilmington, MA, USA) following the method by Carlson et al. (1985) with modifications. TPA (2.5 μg in 10 μL ethanol) was applied topically to both surfaces of the right ear [32]. Ten minutes later, test substances dissolved in a 1:1 ethanol-acetone mixture (10 μL) were applied to the same ear. The left ear served as a control, receiving ethanol followed by the solvent mixture. After four hours, mice were euthanized via CO_2_ inhalation, and 7 mm diameter ear plugs were collected. Edema was measured as ratio of the weight difference between left and the right ear. Percent inhibition (EI%) was calculated using the formula: EI% = 100 − (B × 100)/A, where A represents edema induced by TPA alone and B is the edema induced by TPA plus treatment with 0.1, 0.32, 0.56 and 1 μmol/ear of compounds **1, 2**, or **2b**, respectively. In parallel, an indomethacin curve was included as a positive control. All procedures were approved by the CICUAL-IQ Institutional Ethics Committee (CICUAL-IQ-004-17).

### 4.10. Mieloperoxidase Activity

MPO activity was measured in ear biopsies 4 h post-TPA administration using an adapted protocol of Bradley et al., 1982 and Suzuki et al., 1983 [33,34]. Samples were homogenized in 1.0 mL of PBS (80 mM, pH 5.4) containing 0.5% HTAB, followed by three freeze-thaw cycles and sonication. After centrifugation (13,400× *g*, 15 min, 4 °C), supernatants (10 μL) were incubated in a 96-well plate with 180 μL of HTAB-free PBS at 37 °C. The reaction was initiated with 20 μL (0.017%) H_2_O_2_ were added to each well in the plate at 37 °C. After, 20 μL TMB in 50% aqueous DMF, then halted after 5 min with 2 M H_2_SO_4_. MPO activity was quantified colorimetrically at 450 nm and expressed as percentage inhibition relative to control samples. In parallel, an indomethacin curve was included as a positive control.

### 4.11. Molecular Docking Analysis

Molecular docking analyses were performed using Schrodinger Maestro 13.1 software (Schrödinger Inc., New York, NY, USA). X-ray crystallography structures of both enzymes were obtained from the RCSB Protein Data Bank [35]. For the analysis of exposure to compounds **1** and **2**, ten simulations were performed for xanthine oxidase (PDB ID: 3NVY) and fifteen simulations for MPO (PDB ID:7LAN). The most suitable conformation was selected based on the in vitro results of the compounds’ inhibitory capacity on the proteins.

### 4.12. Statistical Analysis

All data were represented as percentage mean ± SEM. Statistical analyses were made using the GraphPad Prism^®^ software version 8 (GraphPad Software; La Jolla, CA, USA). One-way ANOVA with Dunnett’s post hoc test was used to analyze the results of PCA in mice. The Kruskal–Wallis test was used for in vitro assays such as inhibition of degranulation and inhibition of enzymatic activity.

## 5. Conclusions

Xanthones represent a highly promising chemical scaffold for the treatment of a wide array of chronic inflammatory, allergic, and malignant conditions. Their multitargeting nature, simultaneously regulating cytokines, enzymes, and oxidative stress, makes them superior candidates to traditional single-target therapies, particularly for complex diseases like asthma and skin cancer. Current research highlights jacareubin as a lead molecule for the development of future pharmaceutical and nutraceutical compound derivatives.

## Figures and Tables

**Table 1:** Molecular Docking of Compounds **1** and **2** for Xanthine Oxidase.

Xanthine Oxidase (kJ/mol)				
No.	Title	Tot Q	docking score	glide gscore
4	Compound **2**	−1	−10.251	−10.913
	Residues of XO that interact with compound **2**			
	Residue	Substituent with which it interacts		
1	GLH802	HO-C1		
2	THR1010	HO-C5 and HO-C6		
3	VAL1011	HO-C5		
4	PHE1009	Ring B and C		
5	ARG880	HO-C6		
6	PHE914	Ring C		
No.	Title	Tot Q	docking score	glide gscore
4	Compound **1**	−1	−9.317	−10.019
	Residues of XO that interact with compound **1**			
	Residue	Substituent with which it interacts		
1	GLH802	HO-C1		
2	THR1010	HO-C5 and HO-C6		
3	ARG880	HO-C6		
	PHE1009	Ring B and C		
	PHE914	Ring B and C		

**Table 2:** Molecular Docking of Compounds **1** and **2** for Myeloperoxidase.

Myeloperoxidase (kJ/mol)				
No.	Title	Tot Q	docking score	glide gscore
6	Compound **1**	−1	−6.049	−6.751
	Residues of MPO that interact with compound **1**			
	Residue	Substituent with which it interacts		
1	GLU102	HO-C5		
2	PHE147	HO-C3		
3	ARG239	HO-C6		
No.	Title	Tot Q	docking score	glide gscore
5	Compound **2**	−1	−5.151	−5.814
	Residues of MPO that interact with compound **1**			
	Residue	Substituent with which it interacts		
1	GLN91	HO-C6		
2	HEM601	HO-C6 and Ring C		
3	GLU102	HO-C4		

## Data Availability

The data presented in this study are available on request from the corresponding author due to confidential information that is subject to patenting.

## Supplementary Materials

The following supporting information can be downloaded at: https://www.mdpi.com/article/10.3390/molecules31101666/s1, Figure S1. ^1^H and ^13^C NMR (400 MHz) of compound 1; Figure S2. ^1^H and ^13^C NMR of compound 2 (500 MHz); Figure S3. ^1^H and ^13^C NMR (500 MHz) of compound 1a; Figure S4. ^1^H-NMR (300 MHz) of compound 1c; Figure S5. ^1^H and ^13^C-NMR (500 MHz) of compound 1b; Figure S6. ^1^H and ^13^C-NMR (500 MHz) of compound 2a; Figure S7. ^1^H and ^13^C NMR of compound 2b; Figure S8. Purity of compounds 1 (90%) and 2b (97%) by HPLC-MS; Figure S9. Inhibition of passive cutaneous anaphylaxis in mice. (A) compound 1b to 10 mg/kg; (B) Loratadine to 40 mg/kg. ANOVA one way and Post hoc Dunnet test (* *p* < 0.05%, n = 3–7, SEM); Figure S10. Inhibition of uric acid product of xanthine oxidase by the presence of allopurinol.

## Abbreviations

The following abbreviations are used in this manuscript:

AHR	Airway hyper-responsiveness
BMMCs	Bone marrow-derived mast cells
CHCl_3_	Chloroform
DHE	Dihydroethidium
DMF	N,N-dimethylformamide
DNP-HSA	Dinitrophenyl-human seric albumin
DPI	Diphenyleneiodonium chloride
ED50	Median effective dose
EDTA	Ethylenediaminetetraacetic acid
EGTA	Ethylene glycol-bis(β-aminoethyl ether)-N,N,N′,N′-tetraacetic acid
ERK	Extracellular signal-regulated kinase
FcεRI	High affinity IgE receptor
H_2_SO_4_	Sulfuric acid
HCl	Hydrochloric acid
HO-1	Heme oxygenase-1
HPLC	High performance liquid chromatography
HTAB	Hexadecyltrimethyl-ammonium bromide
IC50	Half maximal inhibitory concentration
IgE	Immunoglobulin type E
JNK	c-Jun N-terminal kinase
LPS	Lipopolysaccharide
MAPKs	Mitogen-activated protein kinases
MeOH	methanol
MPO	Myeloperoxidase
Na_2_CO_3_	Sodium carbonate
NADPH	Nicotinamide adenine dinucleotide phosphate
NBT	Nitrotetrazolium blue
NF-κB	Nuclear factor kappa-light-chain-enhancer of activated B cells
Nrf2	Nuclear factor erythroid 2-related factor 2
OVA	Ovalbumin
PBS	Phosphate-buffered saline
PCA	Passive cutaneous anaphylaxis
ROS	Reactive oxygen species
SEM	Standard error of the mean
SOD	Superoxide dismutase
Syk	Spleen tyrosine kinase
TMB	3,3′,5,5′,tetramethyl-benzidine
TPA	12-O-tetradecanoylphorbol-13-acetate
X-V	Xanthone V
