# Supplementary material for: Jacareubin Derivatives Increase Their Anti-Allergic Activity
Source: Molecules. 2026 May 15;31(10):1666. doi: 10.3390/molecules31101666 (PMC13209822; doi:10.3390/molecules31101666)
Supplement: Supplementary file 1 [file molecules-31-01666-s001.zip › molecules-4265077-supplementary.pdf]

Supplementary figures.

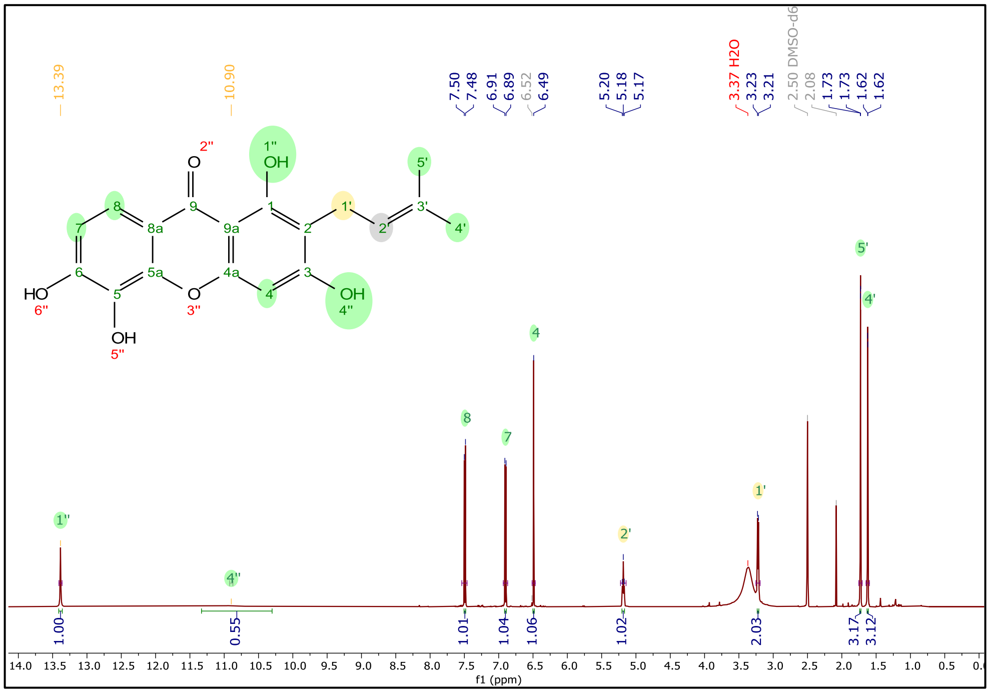

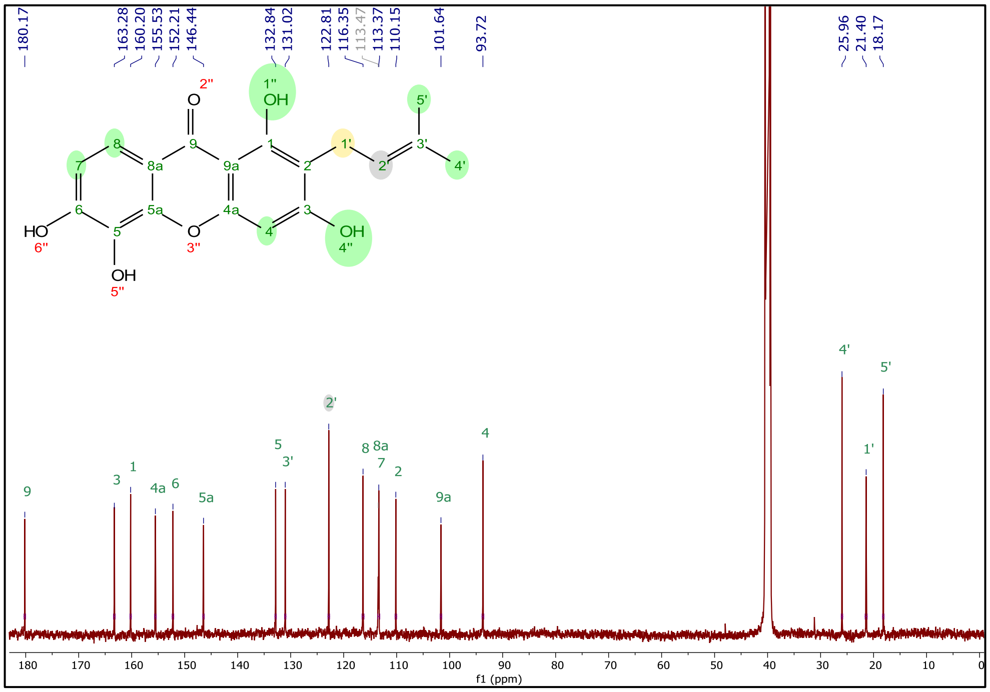

Supplemental Figure S1. ^{1}H and ^{13}C NMR (400 MHz) of compound (1).

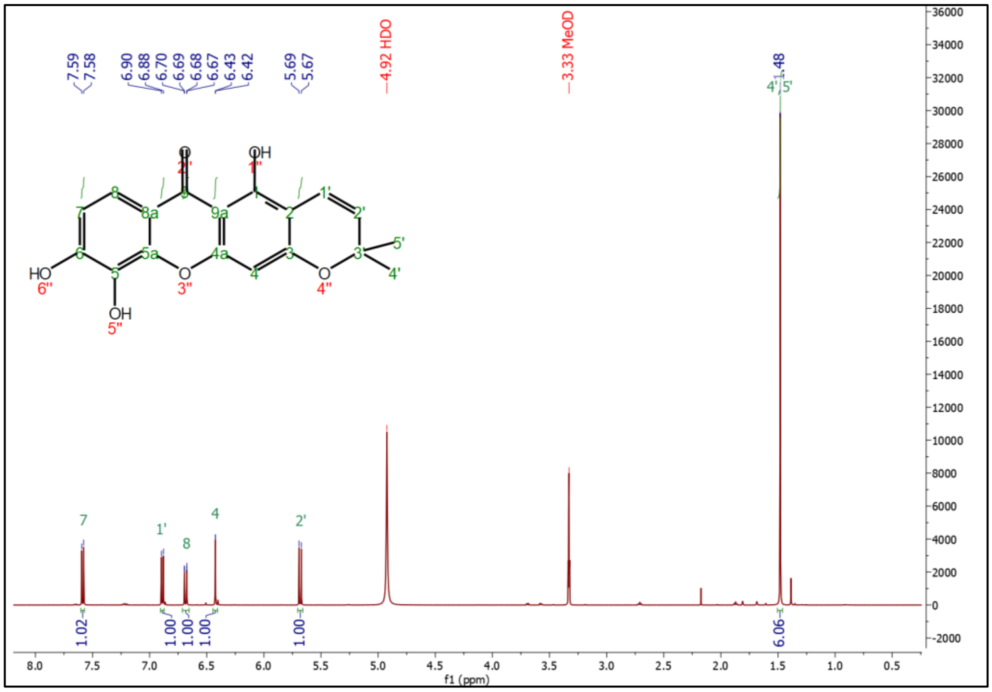

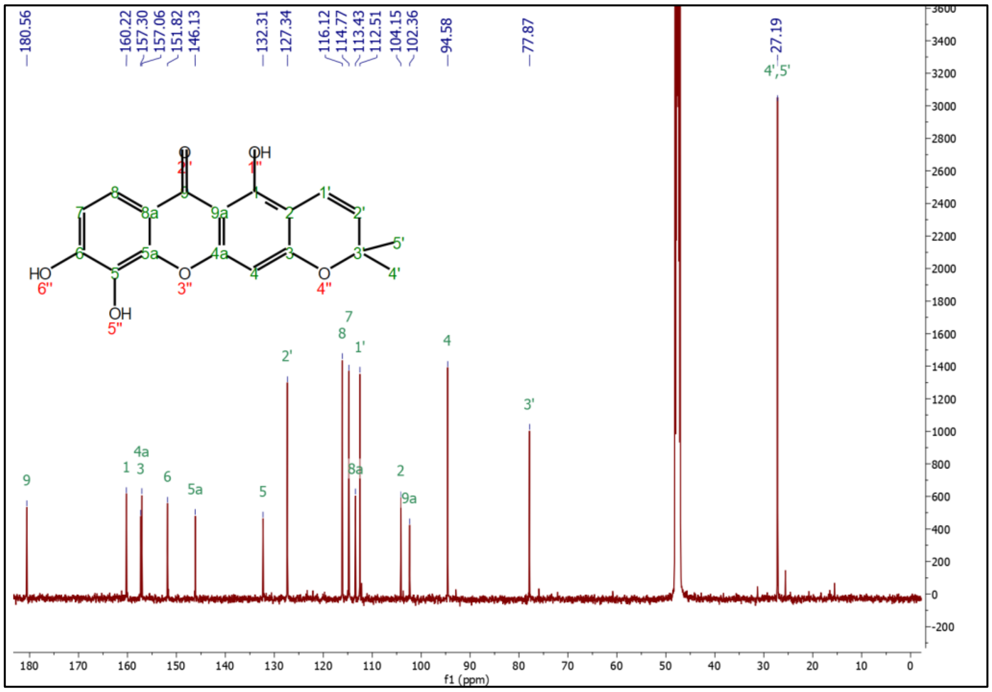

Supplemental Figure S2. ^{1}H and ^{13}C NMR of compound (2) (500 MHz).

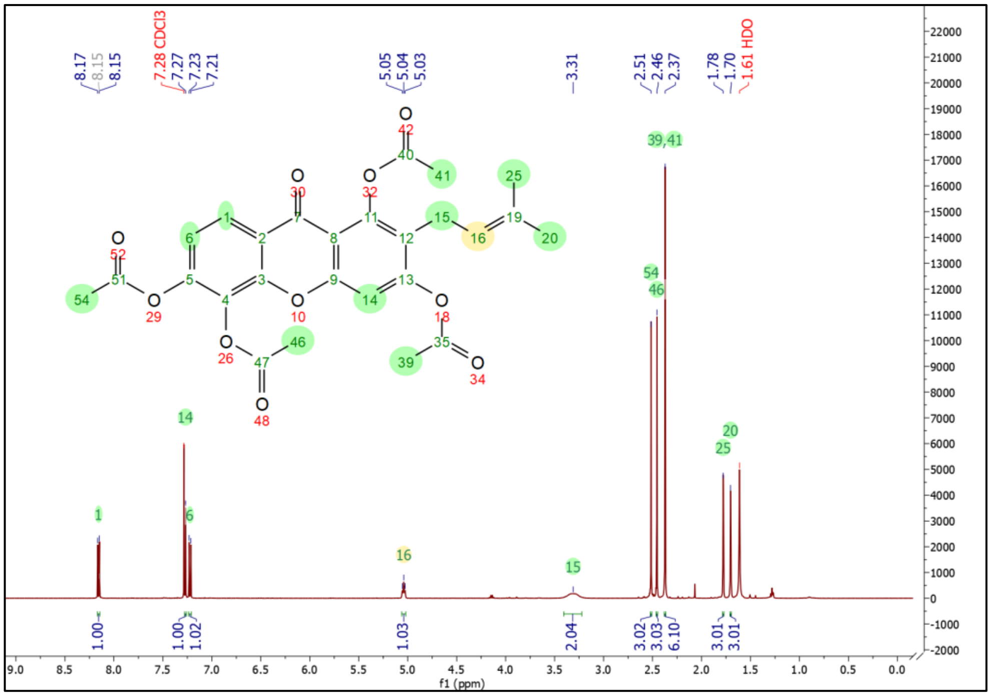

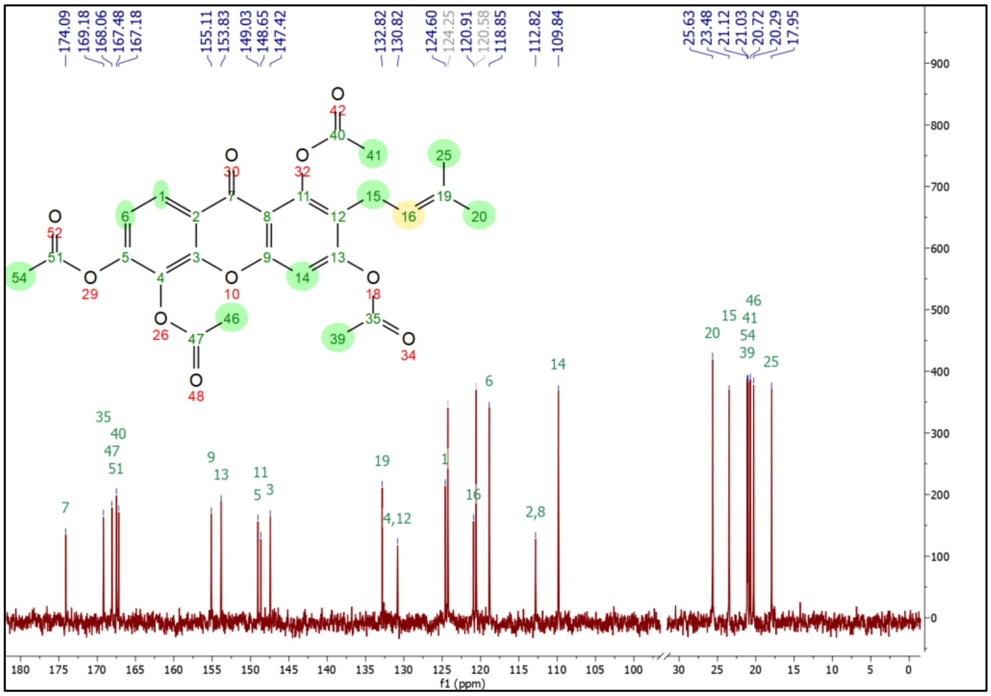

Supplemental Figure S3. ^{1}H and ^{13}C NMR (500 MHz) of compound (1a) .

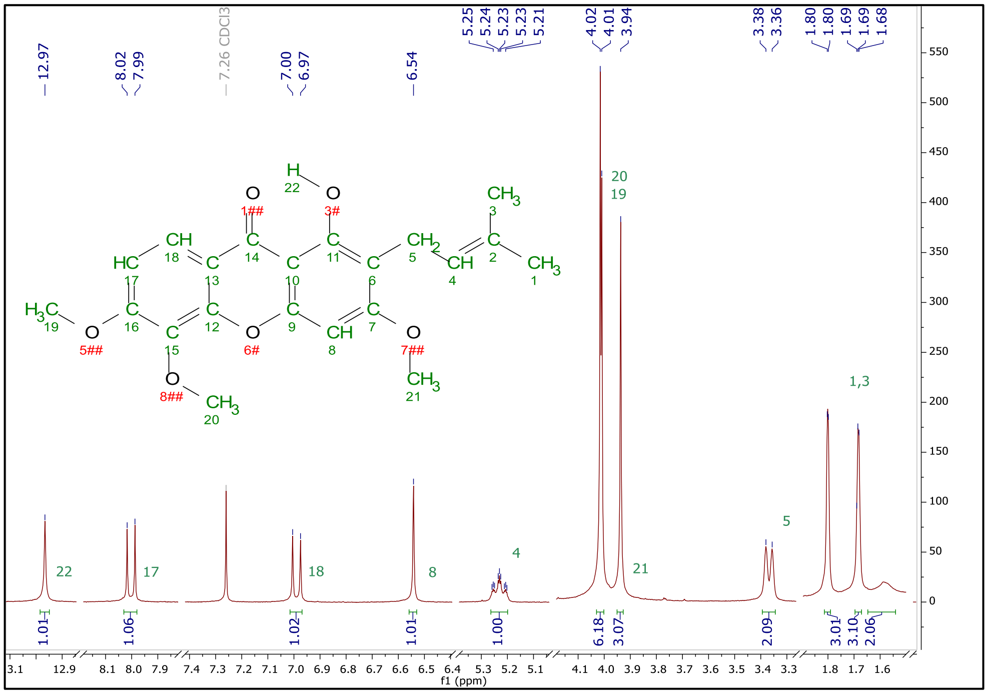

Supplemental Figure S4. ^{1}H-NMR (300 MHz) of compound (1c).

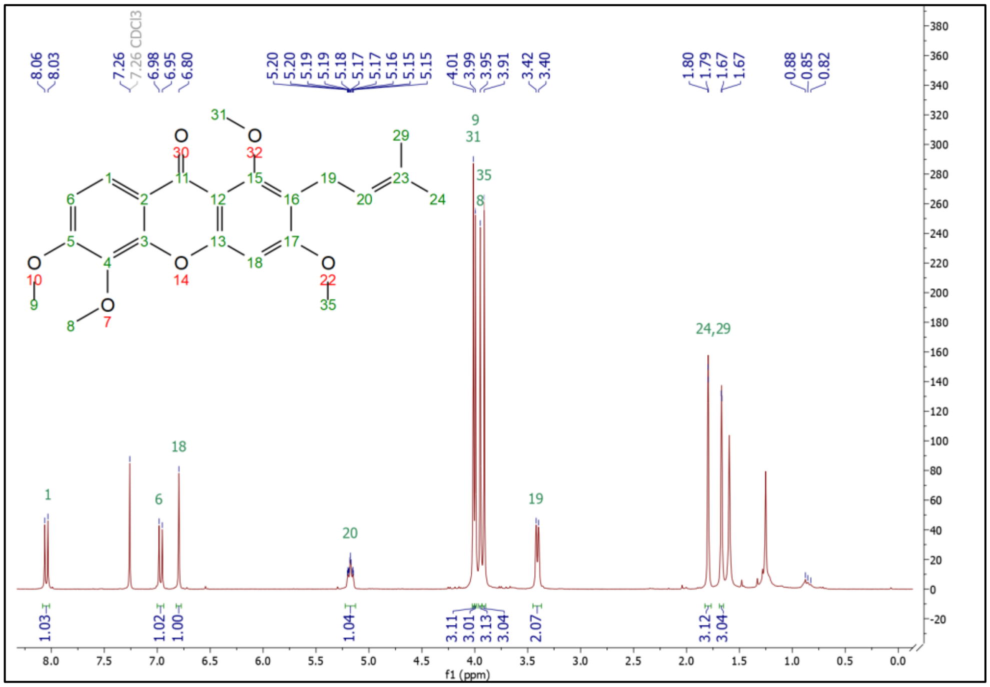

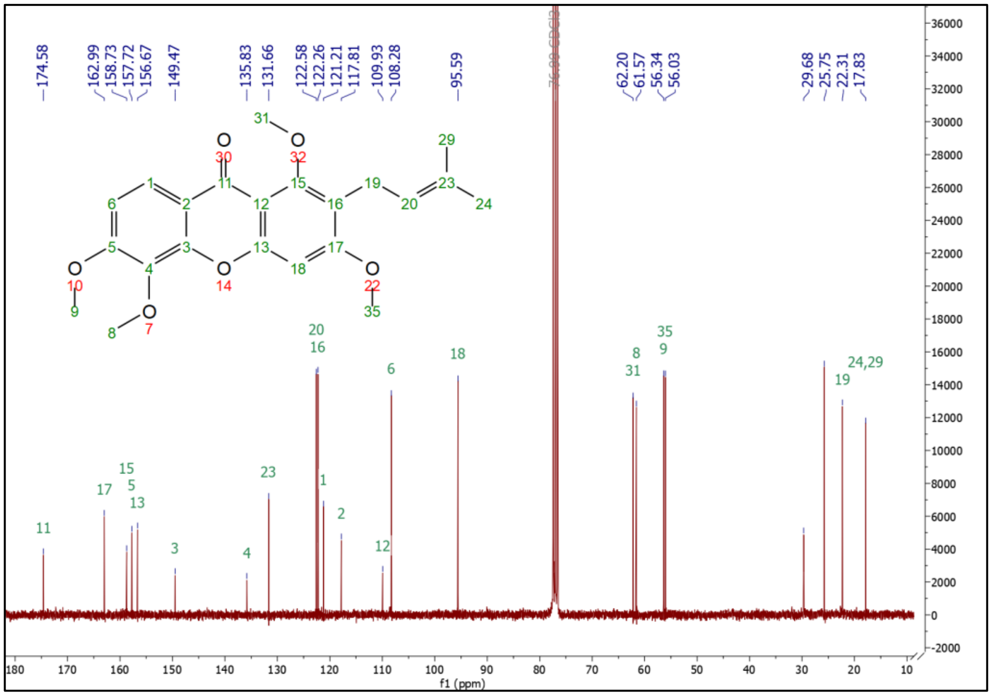

Supplemental Figure S5. ^{1}H and ^{13}C-NMR (500 MHz) of compound (1b).

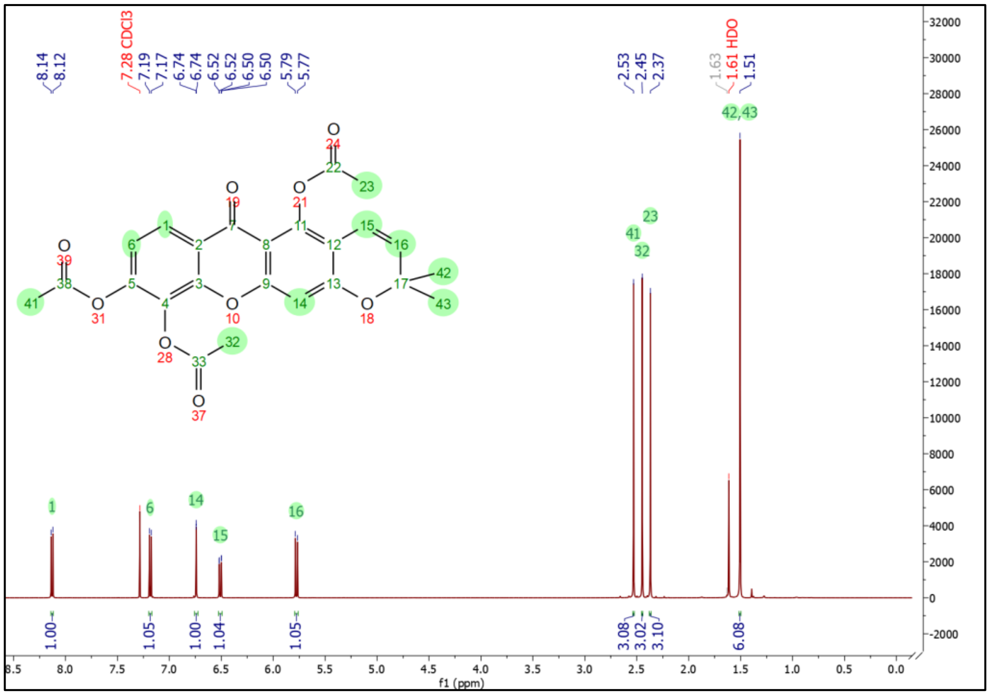

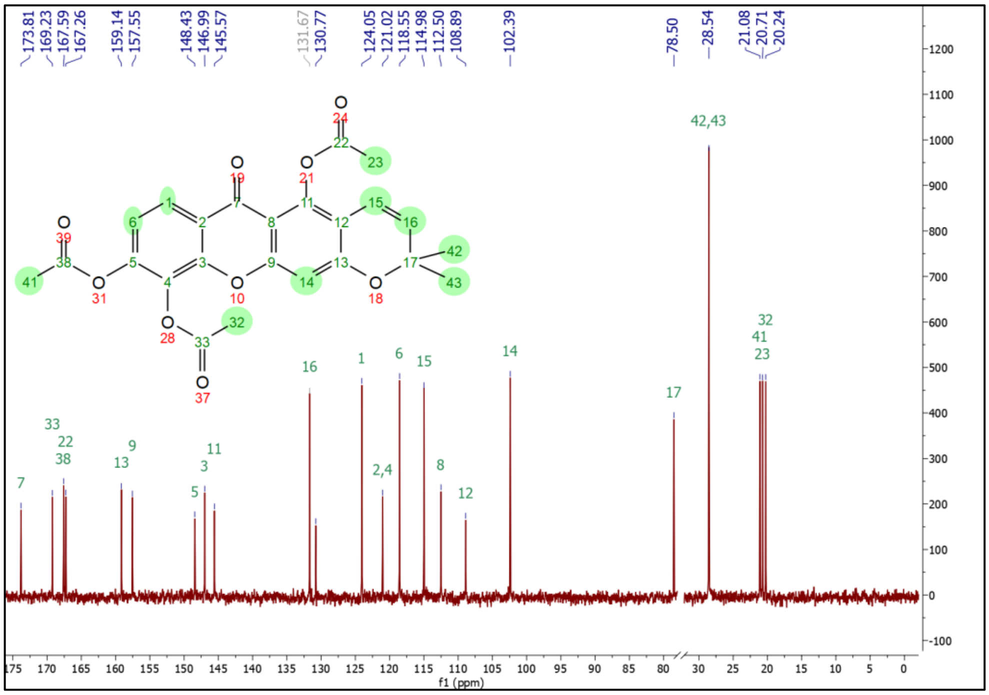

Supplemental Figure S6. ^{1}H and ^{13}C-NMR (500 MHz) of compound (2a).

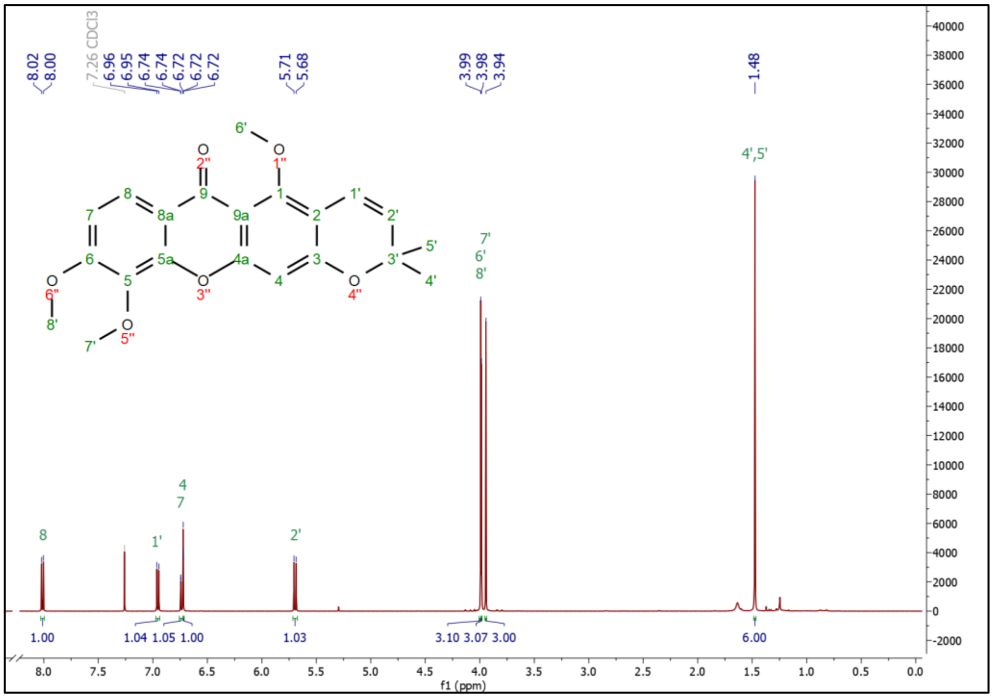

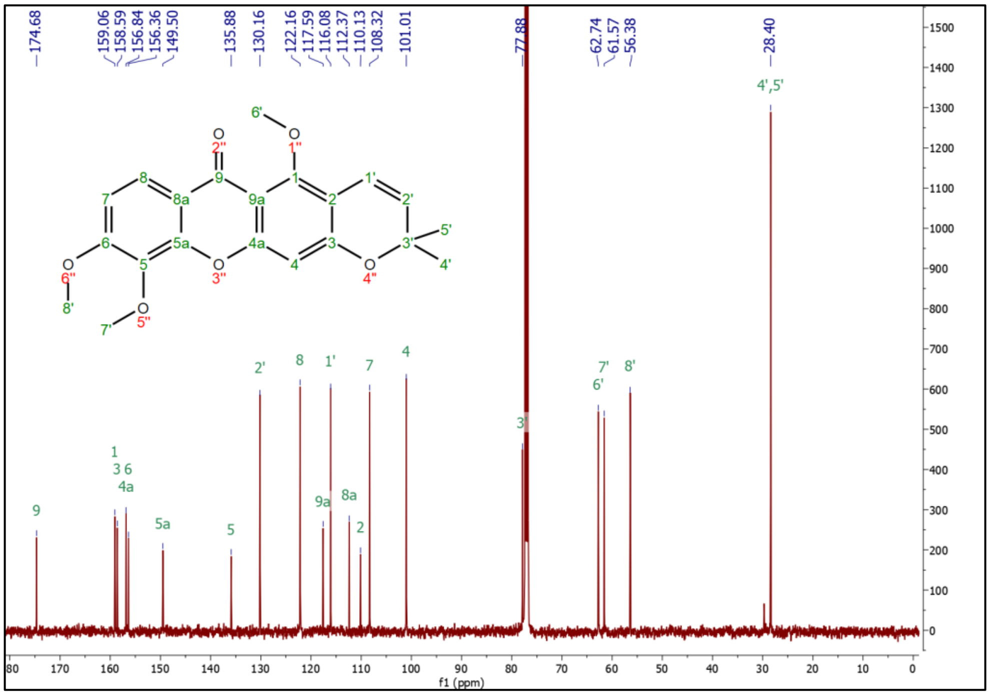

Supplemental Figure S7. ^{1}H and ^{13}C NMR of compound (2b).

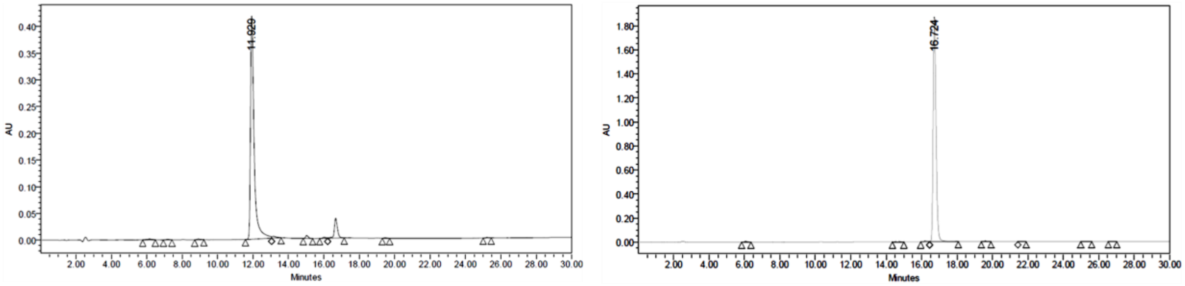

Supplemental Figure S8. Purity of compounds (1) (90%) and (2b) (97%) by HPLC-MS.

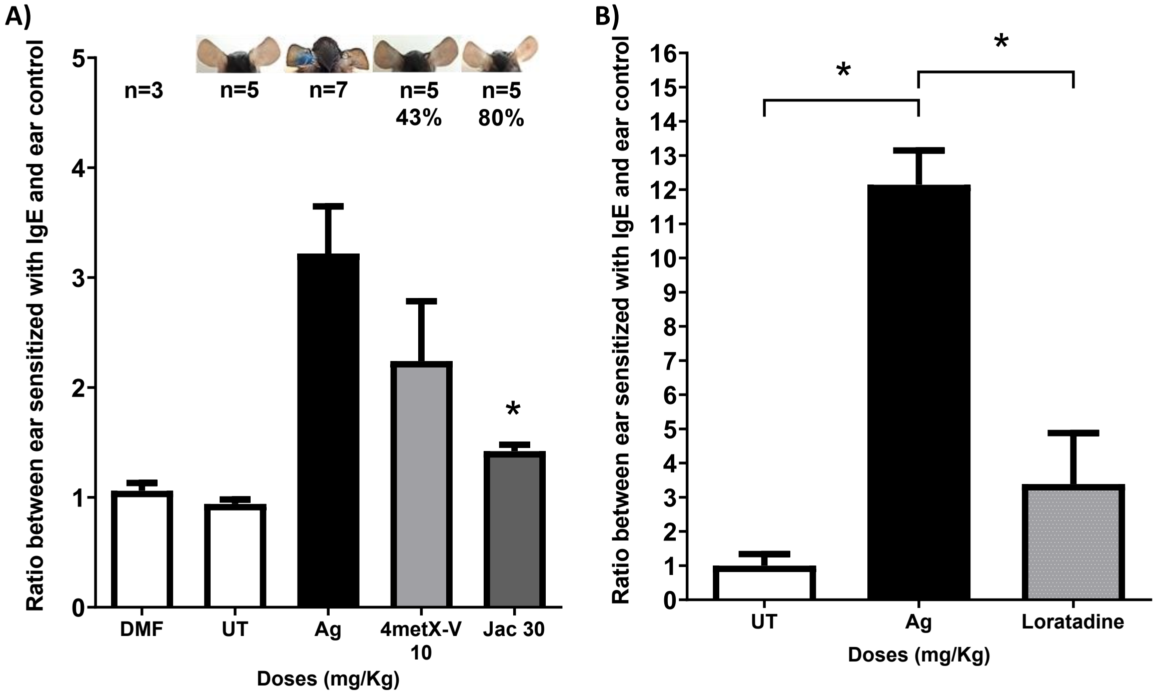

Supplemental Figure S9. Inhibition of passive cutaneous anaphylaxis in mice. A) compound (1b) to 10 mg/Kg; B) Loratadine to 40 mg/Kg. ANOVA one way and Post hoc Dunnet test (*p < 0.05%, n = 3 – 7, SEM).

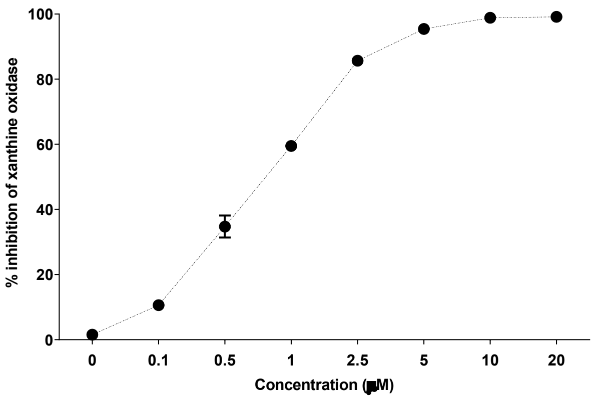

Supplemental Figure S10. Inhibition of uric acid product of xanthine oxidase by the presence of allopurinol (n = 3, SEM).
